# 3*β*,23-Dihydroxy-12-ene-28-ursolic Acid Isolated from *Cyclocarya paliurus* Alleviates NLRP3 Inflammasome-Mediated Gout via PI3K-AKT-mTOR-Dependent Autophagy

**DOI:** 10.1155/2022/5541232

**Published:** 2022-01-10

**Authors:** Dongxiao Lou, Xiaogai Zhang, Cuihua Jiang, Fang Zhang, Chao Xu, Shengzuo Fang, Xulan Shang, Jian Zhang, Zhiqi Yin

**Affiliations:** ^1^Department of Endocrinology, Nanjing Lishui District Hospital of Traditional Chinese Medicine, Nanjing 211200, China; ^2^Laboratory of Translational Medicine, Jiangsu Province Academy of Traditional Chinese Medicine, Nanjing 210028, Jiangsu, China; ^3^Department of TCMs Pharmaceuticals, School of Traditional Chinese Pharmacy, China Pharmaceutical University, Nanjing 211198, Jiangsu, China; ^4^Department of Rheumatology and Immunology, Jiangsu Province Academy of Traditional Chinese Medicine, Nanjing 210028, Jiangsu, China; ^5^College of Forestry, Nanjing Forestry University, Nanjing 210037, Jiangsu, China

## Abstract

Gout is regarded as a painful inflammatory arthritis induced by the deposition of monosodium urate crystals in joints and soft tissues. Nucleotide-binding oligomerization domain (NOD)-like receptor family pyrin domain-containing 3 (NLRP3) inflammasome-mediated IL-1*β* production plays a crucial role in the pathological process of gout. *Cyclocarya paliurus* (CP) tea was found to have an effect on reducing the blood uric acid level of people with hyperuricemia and gout. However, its medicinal ingredients and mechanism for the treatment of gout are still unclear. Thus, this study was designed to investigate the effects of the active triterpenoids isolated from *C. paliurus* on gout and explore the underlying mechanism. The results showed that compound **2** (3*β*,23-dihydroxy-12-ene-28-ursolic acid) from *C. paliurus* significantly decreased the protein expression of IL-1*β*, caspase-1, pro-IL-1*β*, pro-caspase-1, and NLRP3. Furthermore, the production of ROS in the intracellular was reduced after compound **2** treatment. However, ROS agonist rotenone remarkably reversed the inhibitory effect of compound **2** on the protein expression of NLRP3 inflammasome. Additionally, the expression level of LC3 and the ratio of LC3II/LC3I were increased, but the expression level of p62 was suppressed by compound **2** whereas an autophagy inhibitor 3-methyladenine (3-MA) significantly abolished the inhibitory effects of compound **2** on the generation of ROS and the protein expression of NLRP3 inflammasome. Moreover, compound **2** could ameliorate the expression ratio of p-PI3K/PI3K, p-AKT/AKT, and p-mTOR/mTOR. Interestingly, mTOR activator MHY-1485 could block the promotion effect of compound **2** on autophagy regulation and inhibitory effect of compound **2** on induction of ROS and IL-1*β*. In conclusion, these findings suggested that compound **2** may effectively improve NLRP3 inflammasome-mediated gout via PI3K-AKT-mTOR-dependent autophagy and could be further investigated as a potential agent against gout.

## 1. Introduction

Gout is now regarded as an inflammatory arthritis because of the formation and deposition of monosodium urate (MSU) crystals in the joints and soft tissues [1–3]. Recently, the incidence of gout is gradually increasing and shows a low-aging tendency, affecting over 3% of adults in the USA [4]. Chinese Rheumatology Data Center reports that the number of patients suffering from gout in China will reach 100 million by 2021. Gout not only induces damage and degeneration of the joints of the patient, but also causes deformity and disability [5]. In addition, it is also an independent risk factor for many diseases such as metabolic syndrome and cardiovascular diseases [6–9].

With the deeper understanding of the pathological mechanism for gout, the NLRP3 inflammasome activation induced by MSU crystals is found to play a key role in gout development [10, 11]. NLRP3 inflammasome is the cytosolic multiprotein complex composed of NLRP3 and apoptosis-associated speck-like protein containing a caspase recruitment domain (ASC) and pro-caspase-1 [11]. NLRP3 inflammasome activation can trigger the assembly of NLRP3-ASC-pro-caspase-1, resulting in the activation of pro-caspase-1 to produce active caspase-1. Subsequently, the active caspase-1 converts the precursors pro-IL-1*β* and pro-IL-18 into mature and biologically active IL-1*β* and IL-18, respectively [12]. Importantly, the release of IL-1*β* can induce pro-inflammatory cytokine and chemokine production through interaction with IL-1 receptor (IL-1R), leading to neutrophil infiltration at the site of inflammation accompanied by substantial pain and swelling [13]. Therefore, the inhibition of NLRP3 inflammasome-mediated IL-1*β* production process is a new and beneficial strategy for the treatment of gout.

Accumulating research indicates that NLRP3 inflammasome activation is associated with ROS generation [14]. ROS inhibitors or scavengers can prevent NLRP3 inflammasome activation and IL-1*β* production [15]. Recent studies have shown that autophagy participated in the removal of ROS under oxidative stress condition [16]. Autophagy is an intracellular degradation pathway widely existing in eukaryotic cells and can maintain cell homeostasis by the elimination of unfolded or aggregated proteins, damaged organelles, and invading pathogens [17, 18]. Thus, the ROS removal via autophagy regulation can prevent aberrant activation of NLRP3 inflammasome [19]. In addition, autophagy activation can also be regulated by various signals [20]. For example, the phosphatidylinositide 3-kinase (PI3K)-protein kinase B (AKT)-mammalian target of rapamycin (mTOR) signaling pathway has been recognized to negatively regulate the activation of autophagy [21].

In the clinic, the treatment strategy of gout is to decrease the uric acid level and control the acute inflammation response [22]. However, the first-line drugs used to treat gout show some undesired adverse effects, such as myelosuppressive response of colchicine, gastrointestinal side effects of nonsteroidal anti-inflammatory drugs (NSAIDs), and hypersensitivity reaction of allopurinol [23]. Therefore, discovering safe and effective anti-gout drugs is still an urgent clinical need.

*Cyclocarya paliurus* (Batal) Iljinskaja (CP) is a traditional Chinese medicinal herb with Qingre Jiedu and Shengjin Zhike efficiency recorded in Zhonghua Ben Cao [24]. CP leaves have been widely used in the folk medicine for treating diabetes and hyperlipidemia in China. In 2013, it was approved as new food raw material by the Chinese government [25]. Previous pharmacological studies have shown that CP has anti-hyperglycemic, anti-hyperlipidemic, and anti-inflammation activities [26–28]. In addition, it was reported that the *C. paliurus* tea has an effect on reducing the blood uric acid level of people with hyperuricemia and gout [29]. However, its medicinal ingredients and potential mechanism for the treatment of gout are still unclear. Our previous studies found that the triterpenoid-enriched fraction from CP (CPT) was the main active ingredient with good anti-inflammatory effects [26]. Additionally, the pentacyclic triterpenoid such as asiatic acid was reported to improve oxidative stress to inhibit NLRP3 inflammasome activation and the PI3K-AKT-mTOR pathway to activate autophagy [30, 31]. Therefore, we speculate that CPT may be the effective substances to suppress NLRP3 inflammasome activation via PI3K-AKT-mTOR-dependent autophagy to ameliorate gout. Thus, this study aimed to investigate the efficacy of CPT and their triterpenoids on gout and their potential mechanisms.

## 2. Materials and Methods

### 2.1. Reagents

CPT was prepared according to our previous method [32]. Arjunolic acid (compound **1**), 3*β*,23-dihydroxy-12-ene-28-ursolic acid (compound **2**), cyclocaric acid B (compound **3**), 2*a*,3*a*,23-trihydroxyurs-12-en-28-oic (compound **4**), and oleanolic acid (compound **5**) were obtained from our laboratory with >98% purity, and their isolation methods are seen in Supplementary Materials [32]. The structural identification of the above compounds was determined referring to our previous research, and NMR data are added in Supplementary Materials [31]. Primary antibodies against GAPDH, pro-IL-1*β*, pro-caspase-1, IL-1*β*, caspase-1, NLRP3, LC3, p62, p-PI3K, p-AKT, p-mTOR, PI3K, AKT, and mTOR were purchased from Cell Signaling Technology (Danvers, USA). MTT, PMA, and LPS were supplied by KeyGen Biotech (Nanjing, China). ROS assay kit was supplied by Beyotime Institute of Biotechnology (Shanghai, China). ELISA kit for human IL-1*β* was obtained from R&D Systems (Minneapolis, MN). Rotenone, 3-MA, and MHY-1485 were purchased from Sigma-Aldrich (MO, USA).

### 2.2. MSU Crystal Synthesis

MSU crystals were prepared according to the previously described method [33]. In brief, 1 g of uric acid was approximately dissolved and heated in 200 mL of H_2_O with 6 mL NaOH (1 N), then adjusted to pH 8.9, cooled overnight at 4°C, lastly washed, and dried. Needle-like crystals were recovered and suspended in PBS.

### 2.3. Cell Culture and Treatment

Human acute monocytic leukemia THP-1 cells were obtained from Cell Bank of the Chinese Academic of Sciences. The cells were cultured in RPMI-1640 medium with 10% fetal bovine serum, 100 U/mL penicillin, and 100 *μ*g/mL streptomycin under a humidified atmosphere of 5% CO_2_ at 37°C. Cells were stimulated with phorbol 12-myristate 13-acetate (PMA) (100 ng/mL) for 24 h to differentiate into macrophages. These differentiated cells were washed three times with PBS and treated with CPT or triterpenoids for 24 h, then primed with LPS (500 ng/mL) for 3 h, and finally stimulated with MSU (250 *μ*g/mL) for 4 h. Cells were treated with rotenone (10 *μ*M) for 2 h, 3-MA (5 mM) for 1 h, or MHY-1485 (10 *μ*M) for 0.5 h, respectively, before the end of the experiment.

### 2.4. Cell Viability Assay

The effects of CPT and triterpenoids on cell viability were evaluated by the MTT test. THP-1 cells were seeded in 96-well plates with 1 × 10^4^/well and treated with different concentrations of CPT (0, 10, 15, 20, 25, and 30 *μ*g/mL) or triterpenoids (0, 1, 5, 10, 20, 30, 40, and 50 *μ*M) for 24 h. Then, 20 *μ*L MTT (5 mg/mL dissolved in PBS) was added to each well and incubated at 37°C for 4 h. Finally, the supernatants were removed and formazan crystals were dissolved with 150 *μ*L DMSO. The absorbance was detected at 490 nm.

### 2.5. ELISA

After different stimulation methods, the cell supernatants were collected, and the IL-1*β* level was determined by the ELISA kits according to the manufacturer's instructions.

### 2.6. Intracellular ROS Measurement

Cells were cultured in a 6-well plate, treated with various stimulations. Cells were harvested and incubated with 100 *μ*M DCFH-DA attenuated with serum-free medium for 20 min at 37°C in the dark and then washed three times with cold PBS. The images were analyzed using a microscope (Axio Primo Vert.A1, Carl Zeiss, Gottingen, Germany).

### 2.7. Western Blot Assay

Cells with different treatments were washed with PBS and then collected to extract total proteins by adding RIPA lysis buffer for 1 h and centrifuged at 12,000 g at 4°C for 30 min. BCA protein assay kit was used to measure the protein concentration in the supernatants. Then, an equal amount of protein was separated with 10% SDS-PAGE and transferred to PVDF membranes. After being blocked with 5% nonfat milk in TBST buffer, membranes were incubated with specific primary antibodies against GAPDH, pro-IL-1*β*, pro-caspase-1, IL-1*β*, caspase-1, NLRP3, LC3, p62, p-PI3K, p-AKT, p-mTOR, PI3K, AKT, and mTOR overnight at 4°C. These membranes were then washed three times with TBST and incubated by HRP-conjugated secondary antibodies for 1 h at 37°C. The signals were analyzed using the ECL chemiluminescence detection system.

### 2.8. Immunofluorescence Detection

After being treated with various stimulation methods, cells were washed three times with PBS, then fixed with 4% paraformaldehyde at room temperature for 15 min, and washed again with PBS. Subsequently, cells were treated with 0.5% Triton X-100 for 5 min, washed with PBS again, and then treated with 5% FBS for 60 min. Rabbit anti-LC3 was used for immunofluorescence analysis, and goat anti-rabbit FITC-conjugated antibodies were used as the secondary antibody. The positive area was obtained with a microscope.

### 2.9. Statistical Analysis

All data obtained from three independent experiments were expressed as the mean ± SD and analyzed by one-way analysis of variance (ANOVA). The values of *p* < 0.05 were considered statistically significant. All statistical analysis was performed using the software GraphPad Prism 7.

## 3. Results

### 3.1. Effects of CPT and Triterpenoids on Cell Viability

**Figure 1:**
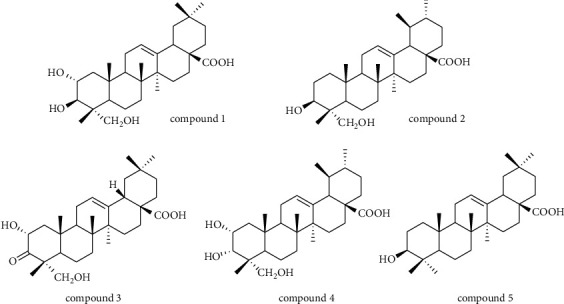
Structures of the triterpenoids from the triterpenoid-enriched fraction of *C. paliurus*. (a) Compound **1**. (b) Compound **2**. (c) Compound **3**. (d) Compound **4**. (e) Compound **5**.

**Figure 2:**
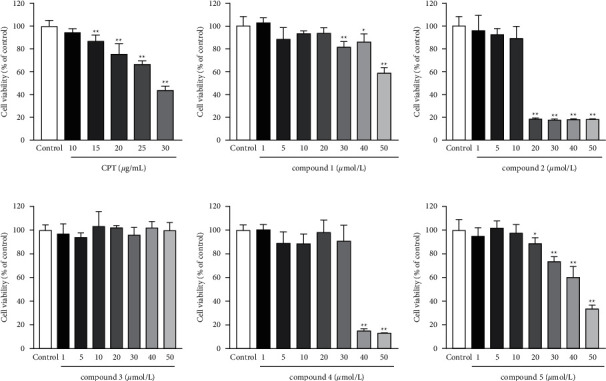
Effects of CPT and triterpenoids on the cell viability of THP-1. The values are expressed as mean ± SD. ^*∗*^*p* < 0.05 and ^*∗∗*^*p* < 0.01 compared with the control group.

The structures of the triterpenoids (compounds **1**–**5**) from the triterpenoid-enriched fraction of *C. paliurus* are shown in Figure 1. To determine the safe concentrations of CPT and triterpenoids, the cell viability assay was conducted by the MTT test. As shown in Figure 2(a)–2(f), CPT (up to 10 ug/mL) did not affect the viability of THP-1. Compound **1** (up to 20 *μ*M), compounds **2** and **5** (up to 10 *μ*M), compound **3** (up to 50 *μ*M), and compound **4** (up to 30 *μ*M) showed no influence on the viability of THP-1. Thus, the safe concentrations were used to evaluate the effects of CPT and triterpenoids on the treatment of gout.

### 3.2. Effects of CPT and Triterpenoids on the Levels of IL-1*β*

**Figure 3:**
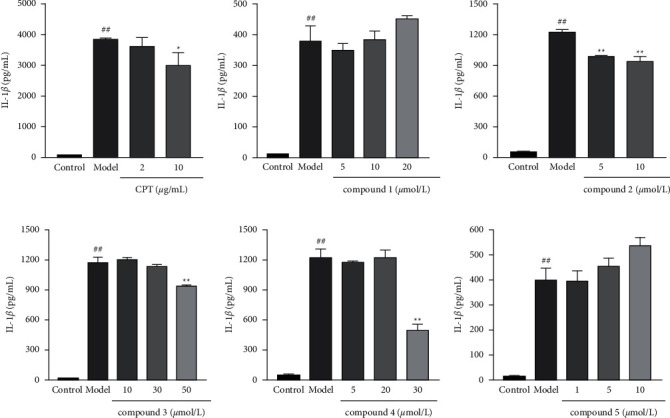
Effects of CPT and triterpenoids on the levels of IL-1*β* in the supernatants of THP-1. The values are expressed as mean ± SD. ^##^*p* < 0.01 compared with the control group. ^*∗*^*p* < 0.05 and ^*∗∗*^*p* < 0.01 compared with the model group.

Generally, NLRP3 inflammasome activation needs two signal steps [34]. Firstly, the priming step is to stimulate pro-IL-1*β* and NLRP3 synthesis through the TLR-MyD88-NF-kB activation caused by LPS or another TLR agonist. The second step is called the activating step, which triggers the caspase-1 activation by the induction of MSU crystals, nigericin, or other stimuli, consequently converting the cytokine precursors pro-IL-1*β* and pro-IL18 into mature and biologically active IL-1*β* and IL-18, respectively. In Figure 3(a)–3(f), the elevated level of IL-1*β* secretion in the model group indicated that LPS evoked the priming step and stimulated MSU to activate NLRP3 inflammasome to successfully establish the gout model in vitro. However, the levels of IL-1*β* were significantly decreased by the treatment with CPT (10 *μ*g/mL), compound **2** (5 *μ*M, 10 *μ*M), compound **3** (50 *μ*M), and compound **4** (30 *μ*M) compared with the model group, whereas compounds **1** and **5** had no effect on the levels of IL-1*β* at different chosen concentrations. Thus, compound **2** was selected for further experiments.

### 3.3. Effects of Compound **2** on the NLRP3 Inflammasome Activation

**Figure 4:**
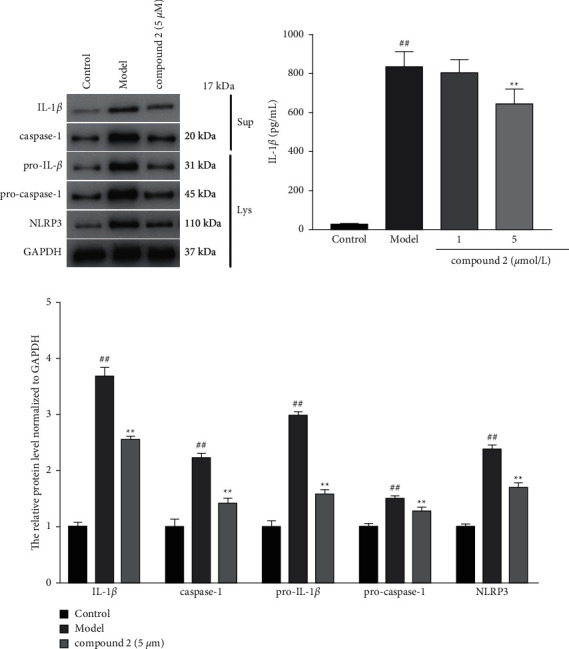
Effects of compound **2** on the expression and activation of the NLRP3 inflammasome. (a) The protein expression levels of IL-1*β* and caspase-1 in the supernatants, as well as pro-IL-1*β*, pro-caspase-1, and NLRP3 in the cell lysates. (b) The levels of IL-1*β* in the supernatants. (c) The relative expression levels of IL-1*β*, caspase-1, pro-IL-1*β*, pro-caspase-1, and NLRP3. The values are expressed as mean ± SD. ^##^*p* < 0.01 compared with the control group. ^*∗*^*p* < 0.05 and ^*∗∗*^*p* < 0.01 compared with the model group.

To explore the effects of compound **2** on the NLRP3 inflammasome activation, the protein expression levels of IL-1*β* and caspase-1 in the supernatants, as well as pro-IL-1*β*, pro-caspase-1, and NLRP3 in the lysates, were determined by the Western blot. As shown in Figures 4(a) and 4(c), compound **2** treatment significantly reduced the protein expression levels of IL-1*β*, caspase-1, pro-IL-1*β*, pro-caspase-1, and NLRP3 compared with the model group. Consistently, ELISA results also exhibited the lower IL-1*β* level in compound **2**-treated group versus the model group (Figure 4(b)). These results suggested that compound **2** may effectively inhibit NLRP3 inflammasome activation.

### 3.4. Effects of Compound **2** on ROS Production

**Figure 5:**
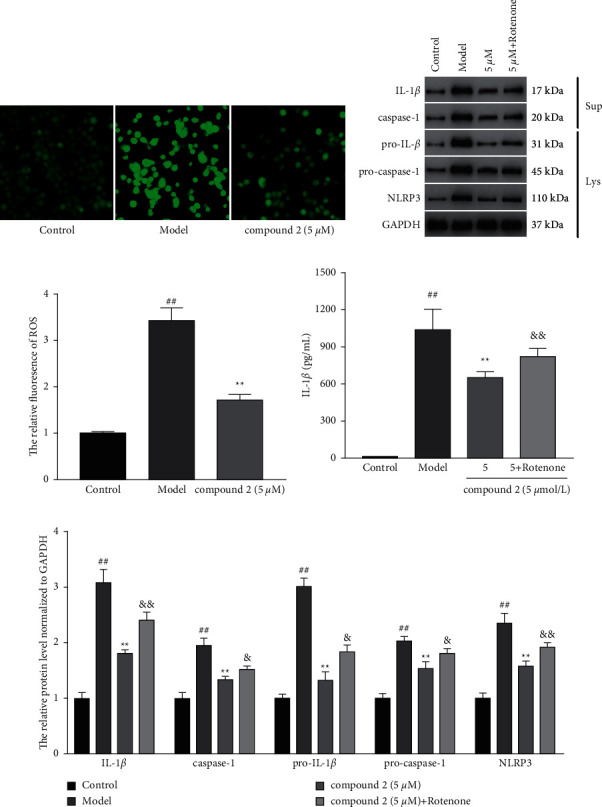
Effects of compound **2** on intracellular ROS production and NLRP3 inflammasome activation. (a) The levels of intracellular ROS. (b) The protein expression levels of IL-1*β* and caspase-1 in the supernatants, as well as pro-IL-1*β*, pro-caspase-1, and NLRP3 in the cell lysates. (c) The relative expression levels of ROS. (d) The levels of IL-1*β* in the supernatants. (e) The relative expression levels of IL-1*β*, caspase-1, pro-IL-1*β*, pro-caspase-1, and NLRP3. The values are expressed as mean ± SD. ^##^*p* < 0.01 compared with the control group. ^*∗∗*^*p* < 0.01 compared with the model group. ^&^*p* < 0.05 and ^&&^*p* < 0.01 compared with compound **2** (5 *μ*M) group.

The activation of NLRP3 inflammasome is closely associated with ROS generation caused by MSU crystals; thus, we explored the effects of compound **2** on ROS generation. As shown in Figures 5(a) and 5(c), the production of ROS was remarkably reduced in compound **2**-treated group compared with the model group. However, the addition of ROS agonist rotenone remarkably reversed the inhibitory effects of compound **2** on the protein expression of IL-1*β*, caspase-1, pro-IL-1*β*, pro-caspase-1, and NLRP3 (Figures 5(b) and 5(e)), as well as the levels of IL-1*β* in the supernatants (Figure 5(d)). These findings indicated that compound **2** might suppress the NLRP3 inflammasome activation by inhibition of ROS production.

### 3.5. Effects of the Compound **2** on Autophagy in THP-1

**Figure 6:**
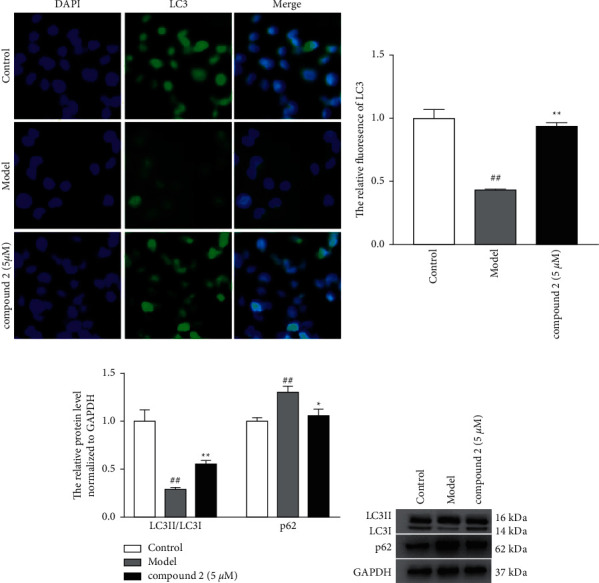
Effects of the compound **2** on the autophagy in THP-1. (a) The protein expression levels of LC3 by immunofluorescence. (b) The relative expression levels of LC3. (c) The relative expression levels of LC3II/LC3I and p62. (d) The protein expression levels of LC3 and p62 by the Western blot. The values are expressed as mean ± SD. ^##^*p* < 0.01 compared with the control group. ^*∗*^*p* < 0.05 and ^*∗∗*^*p* < 0.01 compared with the model group.

**Figure 7:**
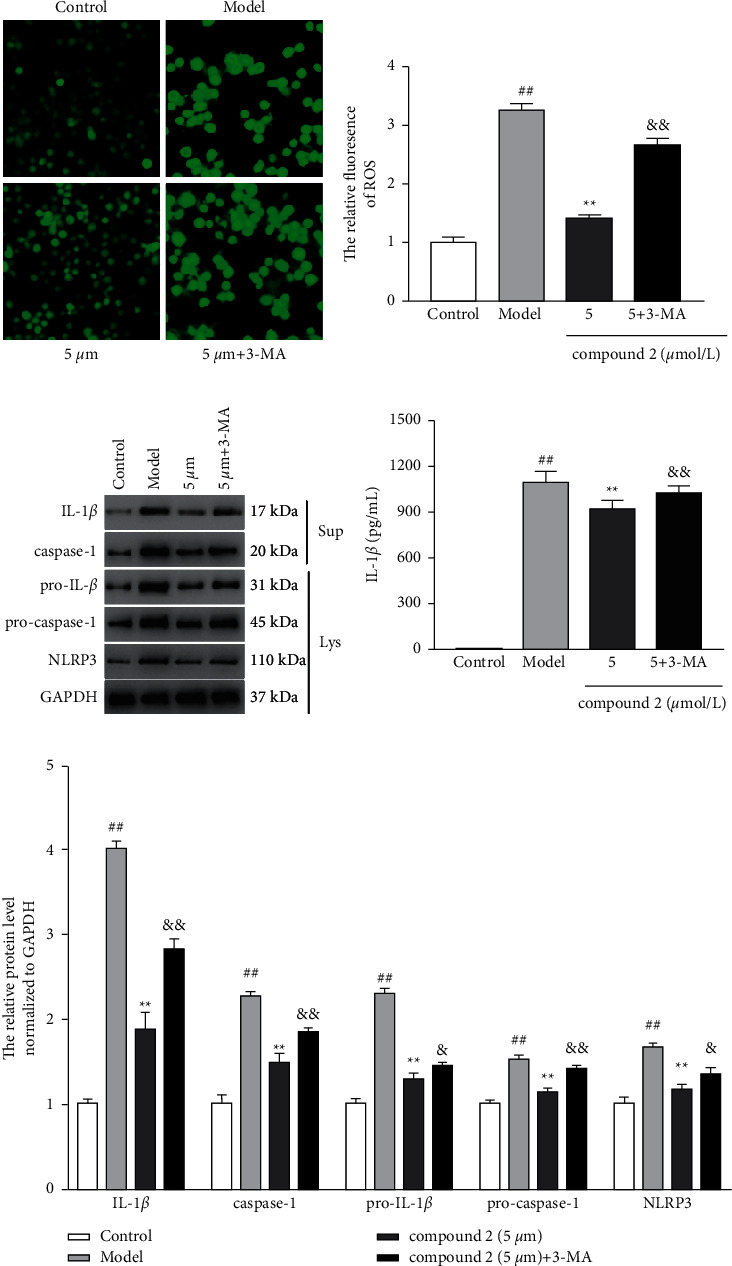
Effects of the autophagy on intracellular ROS production and the expression and activation of the NLRP3 inflammasome. (a) The levels of ROS in the intracellular. (b) The relative expression level of ROS in MSU-induced THP-1. (c) The protein expression levels of IL-1*β* and caspase-1 in the supernatants, as well as pro-IL-1*β*, pro-caspase-1, and NLRP3 in the cell lysates. (d) The levels of IL-1*β* in the supernatants. (e) The relative expression levels of IL-1*β*, caspase-1, pro-IL-1*β*, pro-caspase-1, and NLRP3. The values are expressed as mean ± SD. ^##^*p* < 0.01 compared with the control group. ^*∗∗*^*p* < 0.01 compared with the model group. ^&^*p* < 0.05 and ^&&^*p* < 0.01 compared with compound **2** (5 *μ*M) group.

Recent studies have shown that autophagy participated in the removal of ROS during oxidative stress condition. Thus, we explored the effects of compound **2** on autophagy. As shown in Figures 6(c) and 6(d), compound **2** treatment caused a significant increase in the protein expression ratio of LC3II/LC3I and trigged a remarkable reduction in p62 protein expression. Furthermore, immunofluorescence results also showed the promotion effects of compound **2** on the protein expression of LC3 (Figures 6(a) and 6(b)), whereas an autophagy inhibitor 3-MA significantly abolished the inhibitory effects of compound **2** on ROS generation (Figures 7(a) and 7(b)) and the protein expression of IL-1*β*, caspase-1, pro-IL-1*β*, pro-caspase-1, and NLRP3 (Figures 7(c) and 7(e)). Additionally, the inhibitory effects of compound 2 on the level of IL-1*β* in the supernatants were remarkably reversed by 3-MA (Figure 7(d)). The above results revealed that the inhibitory effect of compound **2** on NLRP3 inflammasome was attributable to promoting autophagy to remove ROS.

### 3.6. Effects of the Compound **2** on the PI3K-AKT-mTOR Signal Pathway

**Figure 8:**
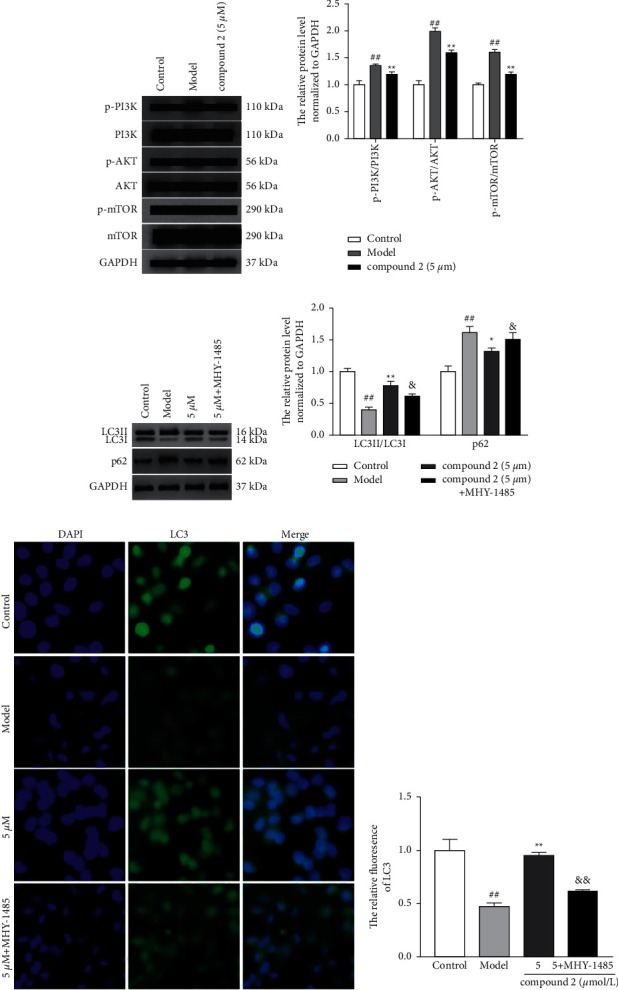
Effects of the compound **2** on the PI3K-AKT-mTOR signal pathway. (a, c) The protein expression levels of PI3K, p-PI3K, AKT, p-AKT, mTOR, p-mTOR, LC3, and p62 by the Western blot. (b, d) The relative expression levels of p-PI3K/PI3K, p-AKT/AKT, p-mTOR/mTOR, LC3II/LC3I, and p62. (e) The protein expression levels of LC3 by immunofluorescence. (f) The relative expression levels of LC3. The values are expressed as mean ± SD. ^##^*p* < 0.01 compared with the control group. ^*∗*^*p* < 0.05 and ^*∗∗*^*p* < 0.01 compared with the model group. ^&^*p* < 0.05 and ^&&^*p* < 0.01 compared with compound **2** (5 *μ*M) group.

**Figure 9:**
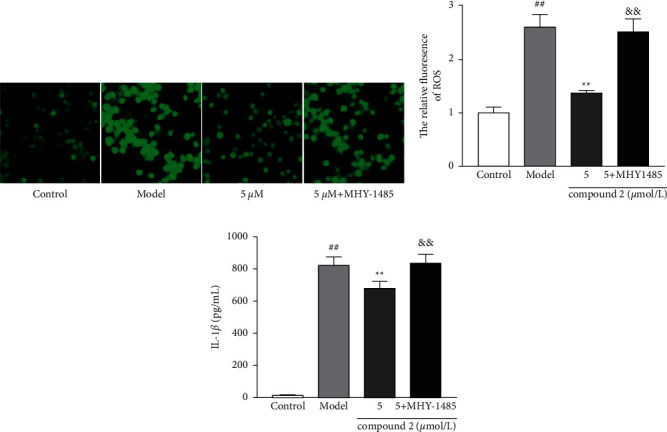
Effects of I3K-AKT-mTOR signal pathway on intracellular ROS and IL-1*β* production. (a) The levels of ROS in the intracellular. (b) The relative expression levels of ROS. (c) The levels of IL-1*β* in the supernatants. The values are expressed as mean ± SD. ^##^*p* < 0.01 compared with the control group. ^*∗∗*^*p* < 0.01 compared with the model group. ^&&^*p* < 0.01 compared with compound **2** (5 *μ*M) group.

**Figure 10:**
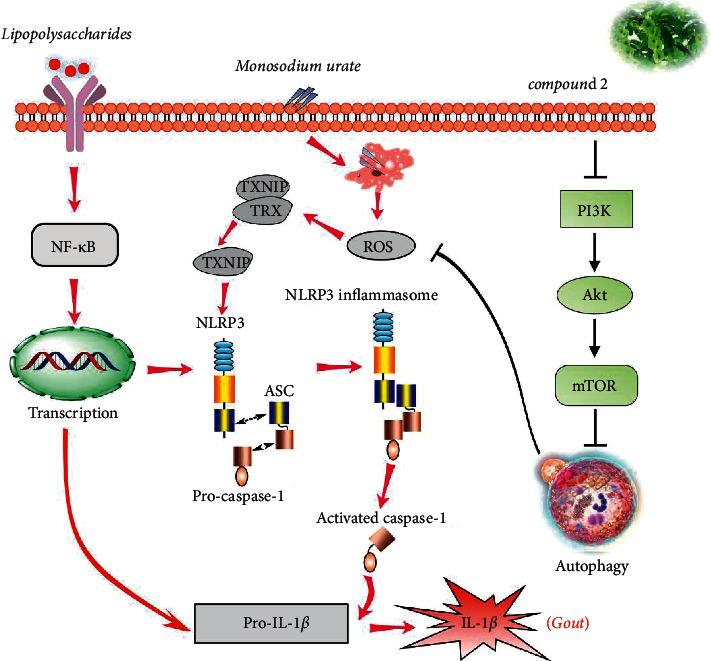
Schematic diagram of the molecular mechanism of compound **2** in treating against gout. LPS could induce upregulation of NLRP3 and pro-IL-1*β* through the activation of transcriptional factor NF-*κ*B. MSU caused release of reactive oxygen species, resulting in the release of thioredoxin interaction protein (TXNIP) from thioredoxin (TRX) and subsequently to NLRP3 inflammasome activation. Besides, activated caspase-1 promoted IL-1*β* production by cleaving pro-IL-1*β*. Compound **2** inhibited the PI3K/AKT/mTOR pathway and induced autophagy activation, which attenuated ROS production and caused the activation of NLRP3 inflammation to be suppressed. In summary, compound **2** alleviated LPS plus MSU-induced gout inflammatory response by inhibiting NLRP3 inflammasome activation via PI3K-AKT-mTOR-dependent autophagy induction.

The PI3K-AKT-mTOR signaling pathway is a classical regulation pathway of autophagy. The inhibition of this signaling pathway can trigger the autophagy development; thus, we explored the effects of compound **2** on PI3K-AKT-mTOR pathway. As shown in Figures 8(a) and 8(b), compound **2** could ameliorate the expression ratio of p-PI3K/PI3K, p-AKT/AKT, and p-mTOR/mTOR, suggesting that compound **2** could suppress activation of PI3K-AKT-mTOR signal pathway. Nonetheless, an mTOR activator MHY-1485 could block the promotion effect of compound **2** on the protein expression ratio of LC3II/LC3I and its inhibitory effect on p62 protein expression (Figures 8(c)–8(f)) and induction of ROS and IL-1*β* in Figures 9(a) and 9(c). These results further suggested that the inhibitory effect of compound **2** on NLRP3 inflammasome may partially depend on preventing the PI3K-AKT-mTOR pathway activation to promote autophagy and ROS removal (Figure 10).

## 4. Discussion

Our present study firstly revealed the effect of CPT on the treatment of gout. Compound **2** was chosen to investigate the potential mechanism of CPT in treating gout. The results indicated that compound **2** may effectively improve NLRP3 inflammasome-mediated gout via PI3K-AKT-mTOR-dependent autophagy. These findings not only broaden the efficacy and potential application of *C. paliurus*, but also provide a candidate compound for anti-gout drugs.

*C. paliurus* as a Chinese medicinal herb has been widely used in the folk medicine for treating diabetes and hyperlipidemia [35]. Our previous study showed the chloroform extract of CP is rich in pentacyclic triterpenoids, which exhibited many biological activities, including anti-hyperglycemia, anti-hyperlipidemia, antioxidant, and anti-inflammation [35, 36]. Moreover, pentacyclic triterpenoids from other plants have been reported to have anti-inflammatory, antitumor, and antioxidant pharmacological effects. For example, ursolic acid as a lipophilic pentacyclic triterpenoid exhibits anti-inflammatory properties via regulation of the NF-*κ*B/NLRP3 inflammasome pathway and ameliorates osteoarthritis [37], celastrol as a pentacyclic triterpenoid quinone methide ameliorates MSU-induced gouty arthritis by inhibiting K63 deubiquitination of NLRP3 [38], and madecassoside is also reported to have anti-inflammation effect on MSU crystal-stimulated gouty arthritis via modulating NLRP3 [39]. Moreover, the NLRP3 inflammasome activation induced by MSU crystals plays a vital role in gout development. Therefore, we speculate that the triterpenoid-enriched fraction from CP (CPT) may be the active ingredients to ameliorate gout through inhibiting NLRP3 inflammasome activation.

Due to an arthritis inflammation response, the patients suffering from gout have poor health-related quality of life. As a first-line treatment for gout, colchicine not only has therapeutic effects but also can cause adverse effects such as myelosuppression and diarrhea [40]. Moreover, the high expensive price of monoclonal antibodies requires a large medical burden [41]. Thus, it is still urgent to develop safe and effective anti-gout drugs to satisfy the clinical need. Recent research reported that some small molecule compounds showed good anti-gout activity [42–44], such as polyphenolic catechin (20 *μ*M), berberine (25 *μ*M), and curcumin (40 *μ*M). Surprisingly, our results showed that compound **2** at lower concentration (5 *μ*M) could suppress the production of IL-1*β*, indicating the better efficacy of compound **2** compared with the above compounds.

In nature, compound **2** has many structural analogs such as ursolic acid, oleanolic acid, asiatic acid, and corosolic acid, belonging to pentacyclic triterpenoids. Ursolic acid at 5 *μ*M concentration could alleviate osteoarthritis by inhibition of the NLRP3 inflammasome in chondrocytes [37]. Similarly, 5 *μ*M concentration of compound **2** showed the inhibitory effect on NLRP3 inflammasome activation in THP-1 cells. The therapeutic dose of ursolic acid in an animal model of osteoarthritis may provide a useful reference for in vivo experiment of compound **2** in the gouty arthritis model. Additionally, ursolic acid possesses various biological activities such as anti-inflammatory, antitumor, and antioxidative [45], suggesting that we may also investigate similar activities of compound **2** in the future. Some previous works on rat models reported the pharmacokinetic studies of structural analogs of compound **2** [46–49]. For example, the bioavailability of asiatic acid was 16.25%, and its oral absorption was better than corosolic acid (0.93%), ursolic acid (8.72%), and oleanolic acid (0.7%), respectively. It was possible because the C23 position of asiatic acid was substituted by the hydroxyl group, leading to an increase in solubility. Similarly, compound **2** has the hydroxyl group at C23 position. Therefore, we speculate that compound **2** may have similar bioavailability to asiatic acid.

Because NLRP3 inflammasome activation has been implicated in the development of several diseases such as gout, asbestosis, metabolic syndrome, and atherosclerosis [50], compound **2** may have a beneficial application for NLRP3 inflammasome-associated diseases based on its inhibitory effect of NLRP3 inflammasome activation in this study.

The production of ROS plays a vital role in NLRP3 inflammasome activation for the development of gout. Currently, several studies have reported that epigallocatechin gallate (EGCG) and procyanidins could inhibit the NLRP3 inflammasome activation by scavenging ROS, but the underlying mechanisms remain unclear [51, 52]. Increasing evidences confirm that autophagy can relieve the NLRP3 inflammasome activation via eliminating the ROS. As excepted, our present study revealed that autophagy-meditated ROS production was responsible for the inhibitory effects of compound **2** on the NLRP3 inflammasome activation.

## 5. Conclusion

Taken together, our study revealed the effect of CPT on the treatment of gout, and compound **2** may effectively improve NLRP3 inflammasome-mediated gout via PI3K-AKT-mTOR-dependent autophagy. These results suggested that compound **2** might be a potential anti-gout agent. However, additional in vivo experiments are necessary to confirm the effect and the underlying mechanism of CPT on gout.

## Data Availability

The data generated and/or analyzed during the study will be available from the corresponding author upon reasonable request.

## Supplementary Materials

Supplementary Materials The methods of the isolation of all compounds, ^1^H and ^13^C NMR results of all compounds, and HPLC analysis results of the purity of all compounds.
