# Supplementary material for: 3β,23-Dihydroxy-12-ene-28-ursolic Acid Isolated from Cyclocarya paliurus Alleviates NLRP3 Inflammasome-Mediated Gout via PI3K-AKT-mTOR-Dependent Autophagy
Source: Evid Based Complement Alternat Med. 2022 Jan 10;2022:5541232. doi: 10.1155/2022/5541232 (PMC8763513; doi:10.1155/2022/5541232)
Supplement: Supplementary Materials — The methods of the isolation of all compounds, 1H and 13C NMR results of all compounds, and HPLC analysis results of the purity of all compounds. [file 5541232.f1.docx]

**Supplementary Materials**

**3*β*, 23-dihydroxy-12-ene-28-ursolic acid isolated from *Cyclocarya paliurus* alleviates NLRP3 inflammasome mediated gout via PI3K-AKT-mTOR-dependent autophagy**

Dongxiao Lou^a,b,c,1^, Xiaogai Zhang^a,1^, Cuihua Jiang^b^, Fang Zhang^d^, Chao Xu^d^, Shengzuo Fang^e^, Xulan Shang^e^, Jian Zhang^b^^[[1]](#footnote-1)^**, Zhiqi Yin^c^^[[2]](#footnote-2)^*

^a^ *Department of Endocrinology, Nanjing Lishui District Hospital of Traditional Chinese Medicine, Nanjing 211200, P.R. China*

^b^*Laboratory of Translational Medicine, Jiangsu Province Academy of Traditional Chinese Medicine, Nanjing 210028, Jiangsu Province, P.R. China*

^c^ *Department of TCMs Pharmaceuticals, School of Traditional Chinese Pharmacy, China Pharmaceutical University**, Nanjing 211198, Jiangsu Province, P.R. China*

^d^*Department of Rheumatology and Immunology,* *Jiangsu Province Academy of Traditional Chinese Medicine, Nanjing 210028, Jiangsu Province, P.R. China*

^e^ *College of Forestry, Nanjing Forestry University, Nanjing, 210037, Jiangsu Province, P.R. China*

^1^ These authors contributed equally: Dongxiao Lou, Xiaogai Zhang

Supplementary material relating to this article is available online, alongside related materials and Figures S1-S14.

1. **Extraction and Isolation**

Air-dried leaves of *Cyclocarya paliurus* (48.5 kg) were extracted three times with 80% ethanol under reflux and evaporated to afford a crude extract (8.3 kg). The extract was suspended in water, then partitioned with chloroform to yield a chloroform-soluble extract (3.6 kg). The chloroform extract was subjected to a decompression silica gel column chromatography and eluted with gradient mixtures of CH_3_Cl-MeOH (100:0→0:100) to yield 5 fractions (Fr.1 - 5). Fr.2 was subjected to a silica gel column, Sephadex LH-20 column and followed by semi-preparative HPLC, together with recrystallization to obtain compounds **1** (1000 mg), **2** (900 mg), **3** (15 mg), **4** (600 mg) and **5** (1500 mg).

1. **NMR data of compounds 1-5**

2.1 arjunolic acid (**1**)

^1^H NMR (C_5_D_5_N , 500 MHz) *δ*_H_: 5.46 (1H, br s, H-12), 4.19 (1H, m, H-2), 3.72 (1H, d, *J* = 10.5 Hz, H-23), 3.60 (1H, s, H-3), 3.29 (1H, d, *J* = 11.0 Hz, H-23), 2.29 (1H, dd, *J* = 12.3, 4.0 Hz, H-18), 1.21 (3H, s, Me-24), 1.08 (3H, s, Me-25), 1.07 (3H, s, Me-26), 1.04 (3H, s, Me-27), 0.99 (3H, s, Me-29), 0.92 (3H, s, Me-30). ^13^C NMR (C_5_D_5_N, 125 MHz) *δ*_C_: 178.6 (C-28), 145.0 (C-13), 123.1 (C-12), 78.3 (C-3), 68.9 (C-2), 66.6 (C-23), 48.2 (C-9), 48.0 (C-5), 47.7 (C-17), 46.7 (C-1), 46.5 (C-19), 43.6 (C-18), 42.3 (C-4), 42.0 (C-14), 39.9 (C-8), 38.4 (C-10), 34.3 (C-21), 33.2 (C-7), 33.2 (C-29), 32.9 (C-22), 30.9 (C-20), 28.3 (C-15), 26.1 (C-27), 23.9 (C-16), 23.8 (C-11), 23.7 (C-30), 18.6 (C-6), 17.6 (C-25), 17.4 (C-26), 14.3 (C-24).

2.2 3β,23-dihydroxy-12-ene-28-ursolic acid (**2**)

^1^H NMR (C_5_D_5_N, 300 MHz) *δ*_H_: 5.52 (1H, br s, H-12), 4.22 (1H, t, *J* = 8.3, 10.1 Hz, H-3), 4.20 (1H, d, *J* = 10.1 Hz, H-23), 3.74 (1H, d, *J* = 10.4 Hz, H-23), 2.63 (1H, d, *J* = 10.9 Hz, H-18), 1.26 (3H, s, Me-24), 1.20, (3H, s, Me-25)1.02 (3H, s, Me-26), 0.99 (3H, s, Me-27), 1.08 (3H, d, *J* = 6.0 Hz, Me-29), 0.95 (3H, d, *J* = 5.0 Hz, Me-30). ^13^C NMR (C_5_D_5_N, 75 MHz) *δ*_C_: 180.7 (C-28), 139.7 (C-13), 126.2 (C-12), 74.0 (C-3), 68.5 (C-23), 54.1 (C-18), 49.1 (C-9), 48.6 (C-17), 48.5 (C-5), 43.3 (C-4), 42.7 (C-14), 40.5 (C-8), 40.0 (C-1), 39.9 (C-20), 39.4 (C-19), 37.7 (C-10), 37.6 (C-22), 33.7 (C-7), 31.6 (C-21), 28.8 (C-15), 28.2 (C-2), 25.4 (C-16), 24.4 (C-11), 24.2 (C-27), 21.8 (C-30), 19.1 (C-6), 17.9 (C-26), 16.6 (C-29), 16.4 (C-25), 13.6 (C-24).

2.3 cyclocaric acid B (**3**)

^1^H NMR (C_5_D_5_N, 500 MHz) *δ*_H_: 5.50 (1H, br s, H-12), 4.55 (1H, dd, *J* = 11.8、4.9 Hz, H-2), 4.12 (1H, d, *J* = 10.6 Hz, H-23), 3.71 (1H, d, *J* = 10.6 Hz, H-23), 2.78 (1H, dd, *J* = 11.8、4.9 Hz, H-18), 1.35 (3H, s, Me-24), 1.22 (3H, s, Me-25), 1.16 (3H, s, Me-26), 1.08 (3H, s, Me-27), 0.97 (3H, s, Me-29), 0.90 (3H, s, Me-30). ^13^C NMR (C_5_D_5_N, 125 MHz) *δ*_C_: 212.8 (C-3), 180.1 (C-28), 144.1 (C-13), 123.3 (C-12), 72.8 (C-2), 66.0 (C-23), 52.4 (C-4), 47.4 (C-5), 46.8 (C-9), 46.1 (C-1), 45.1 (C-19), 43.8 (C-17), 42.5 (C-14), 42.3 (C-18), 39.7 (C-8), 39.6 (C-10), 34.2 (C-21), 33.2 (C-29), 33.1 (C-7), 32.9 (C-22), 30.9 (C-20), 28.3 (C-15), 25.9 (C-27), 25.8 (C-16), 23.7 (C-11), 23.7 (C-30), 18.1 (C-25), 17.9 (C-6), 15.6 (C-26), 13.7 (C-24).

2.4 2a, 3a, 23-trihydroxyurs-12-en-28-oic acid (**4**)

^1^H NMR (C_5_D_5_N, 600 MHz) *δ*_H_: 5.48 (1H, br t, *J* = 3.3 Hz, H-12), 4.30 (1H, ddd, *J* = 3.0, 3.9, 11.6 Hz, H-2), 4.17 (1H, d, *J* = 2.4 Hz, H-3), 3.94 (1H, d, *J* = 10.8 Hz, H-23a), 3.78 (1H, d, *J* = 10.7 Hz, H-23b), 2.63 (1H, d, *J* = 11.3 Hz, H-18), 1.15 (3H, s, CH3-27), 1.08 (3H, s, Me-25), 1.00 (3H, s, Me-24), 0.97 (3H, d, *J* = 6.5 Hz, Me-29), 0.93 (3H, d, *J* = 6.4 Hz, Me-30), 0.88 (3H, s, Me-26). 13C NMR (C_5_D_5_N, 150 MHz) *δ*_C_: 179.9 (C-28), 139.3 (C-13), 125.5 (C-12), 78.9 (C-3), 71.3 (C-23), 66.9 (C-2), 53.5 (C-18), 48.0 (C-9), 48.0 (C-17), 43.5 (C-5), 42.8(C-1), 42.6 (C-14), 41.9 (C-8), 40.1 (C-4), 39.4 (C-19), 39.4 (C-20), 38.4 (C-10), 37.5 (C-22), 33.2 (C-7), 33.1 (C-21), 28.6 (C-15), 24.9 (C-16), 23.8 (C-27), 23.7 (C-11), 21.4(C-30), 18.3 (C-6), 17.8 (C-29), 17.5 (C-25), 17.5 (C-26), 17.2 (C-24).

2.5 oleanic acid (**5**)

^1^H NMR (C_5_D_5_N, 500 MHz) *δ*_H_: 5.51 (1H, br t, *J* = 3.5 Hz, H-12), 3.46 (1H, dd, *J* = 5.5, 11.0 Hz, H-3), 3.32 (1H, dd, *J* = 4.0, 13.5 Hz), 1.31 (3H, s, Me-23), 1.25 (3H, s, Me-24), 1.05 (3H, s, Me-29), 1.04 (3H, s, Me-30), 1.03 (3H, s, Me-25), 0.97 (3H, s, Me-27), 0.93 (3H, s, Me-26). ^13^C NMR (C_5_D_5_N, 125 MHz) *δ*_C_: 180.5 (C-28), 145.3 (C-13), 123.0 (C-12), 78.5 (C-3), 56.3 (C-5), 48.6 (C-9), 47.1 (C-17), 47.0 (C-18), 42.6 (C-19), 42.5 (C-14), 40.2 (C-8), 39.8 (C-4), 39.4 (C-1), 37.8 (C-10), 34.7 (C-21), 33.7 (C-22), 33.7 (C-29), 33.6 (C-7), 31.4 (C-20), 29.2 (C-23), 28.8 (C-15), 28.5 (C-2), 26.6 (C-27), 24.3 (C-16), 24.2 (C-11), 24.2 (C-30). 19.2 (C-6), 17.9 (C-26), 16.9 (C-24), 16.0 (C-25).

1. **Figures of NMR Spectrum and HPLC profile of compounds 1-5**

Fig. S1. ^1^H NMR Spectrum of compound **1** (C_5_D_5_N, 500 MHz)

Fig. S2. ^13^C NMR Spectrum of compound **1** (C_5_D_5_N, 125 MHz)

Fig. S3. HPLC profile of compound **1** using UV detection at 205 nm.

Fig. S4. ^1^H NMR Spectrum of compound **2** (C_5_D_5_N, 300 MHz)

Fig. S5. ^13^C NMR Spectrum of compound **2** (C_5_D_5_N, 75 MHz)

Fig. S6. HPLC profile of compound **2** using UV detection at 205 nm.

Fig. S7. ^1^H NMR Spectrum of compound **3** (C_5_D_5_N, 500 MHz)

Fig. S8. ^13^C NMR Spectrum of compound **3** (C_5_D_5_N, 125 MHz)

Fig. S9. HPLC profile of compound **3** using UV detection at 205 nm.

Fig. S10. ^1^H NMR Spectrum of compound **4** (C_5_D_5_N, 600 MHz)

Fig. S11. ^13^C NMR Spectrum of compound **4** (C_5_D_5_N, 150 MHz)

Fig. S12. HPLC profile of compound **4** using UV detection at 205 nm.

Fig. S13. ^1^H NMR Spectrum of compound **5** (C_5_D_5_N, 500 MHz)

Fig. S14. ^13^C NMR Spectrum of compound **5** (C_5_D_5_N, 125 MHz)


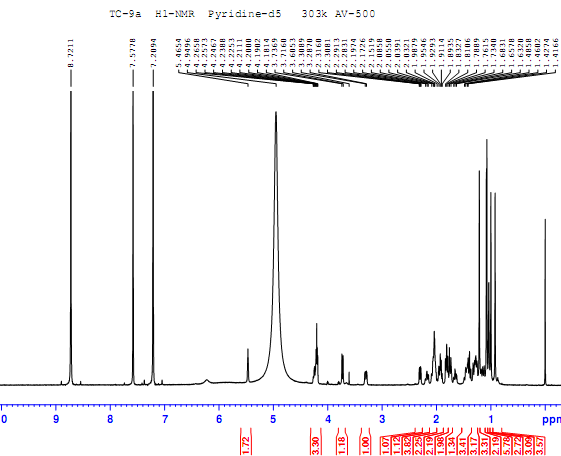


Fig. S1. ^1^H NMR Spectrum of compound **1** (C_5_D_5_N, 500 MHz)


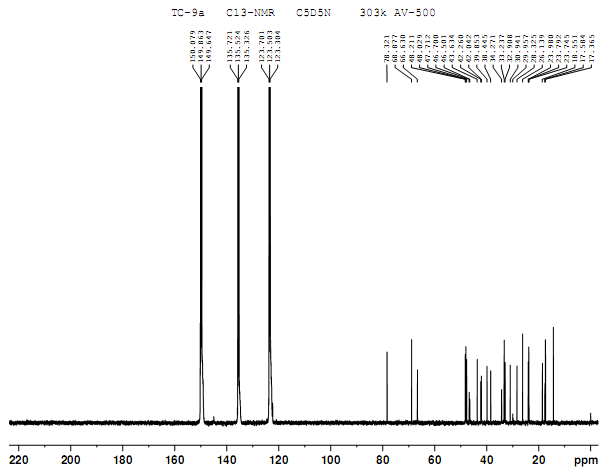


Fig. S2. ^13^C NMR Spectrum of compound **1** (C_5_D_5_N, 125 MHz)


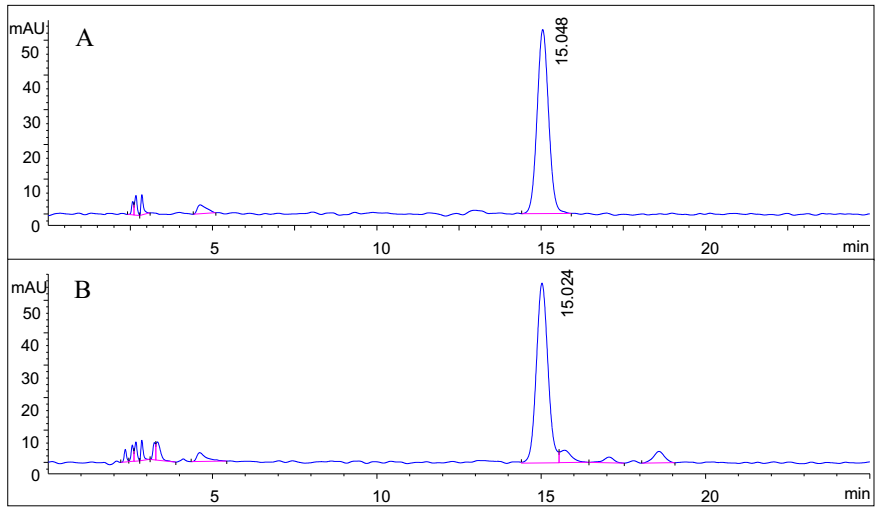


Fig. S3. HPLC profile of compound **1** using UV detection at 205 nm.


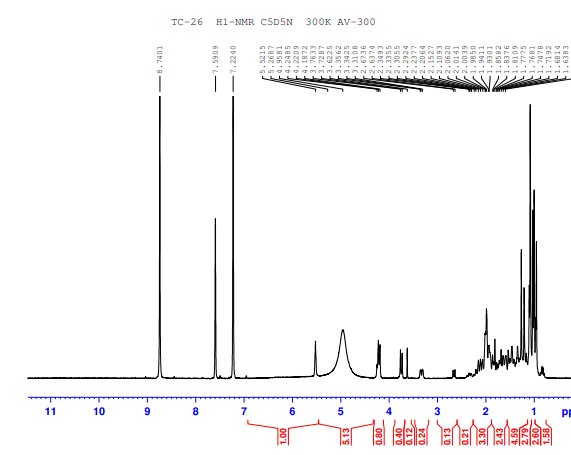


Fig. S4. ^1^H NMR Spectrum of compound **2** (C_5_D_5_N, 300 MHz)


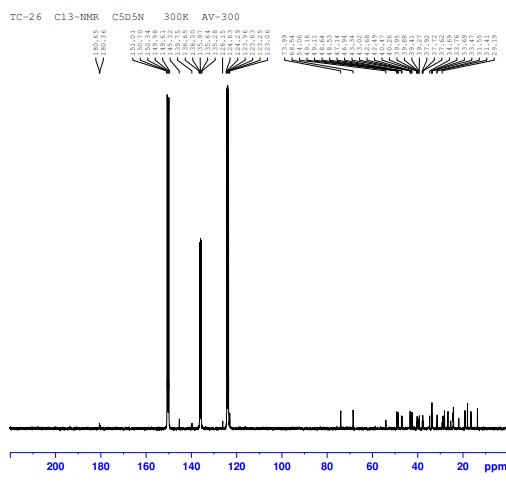


Fig. S5. ^13^C NMR Spectrum of compound **2** (C_5_D_5_N, 75 MHz)


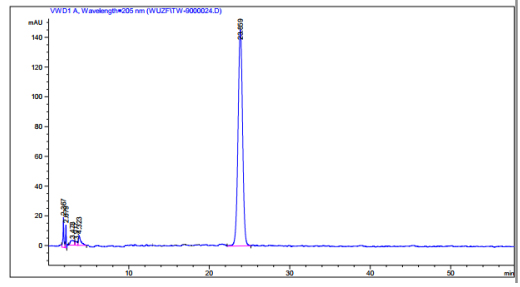


Fig. S6. HPLC profile of compound **2** using UV detection at 205 nm.


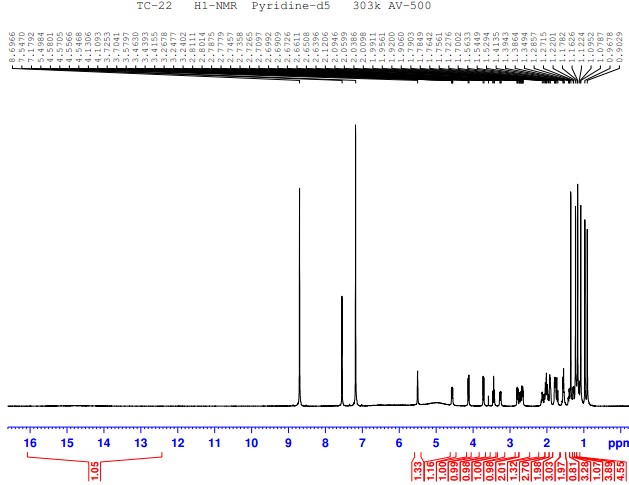


Fig. S7. ^1^H NMR Spectrum of compound **3** (C_5_D_5_N, 500 MHz)


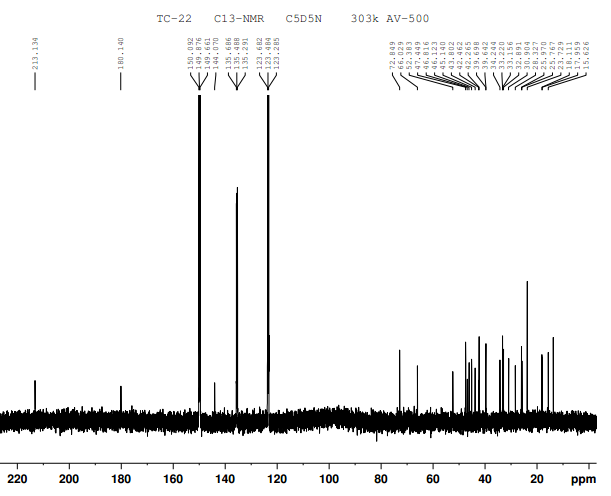


Fig. S8. ^13^C NMR Spectrum of compound **3** (C_5_D_5_N, 125 MHz)


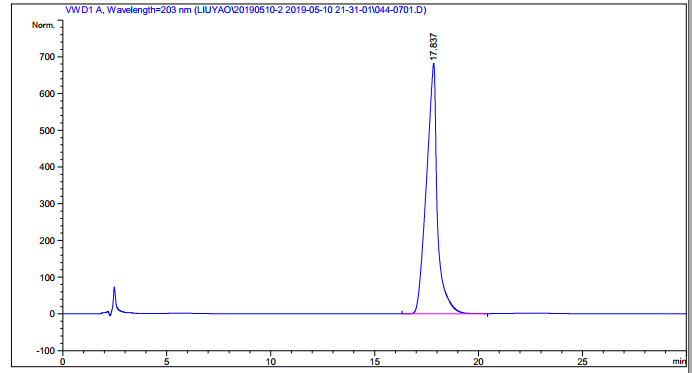


Fig. S9. HPLC profile of compound **3** using UV detection at 205 nm.


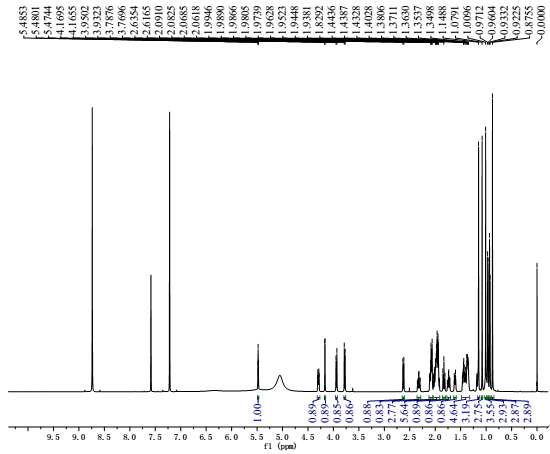


Fig. S10. ^1^H NMR Spectrum of compound **4** (C_5_D_5_N, 600 MHz)


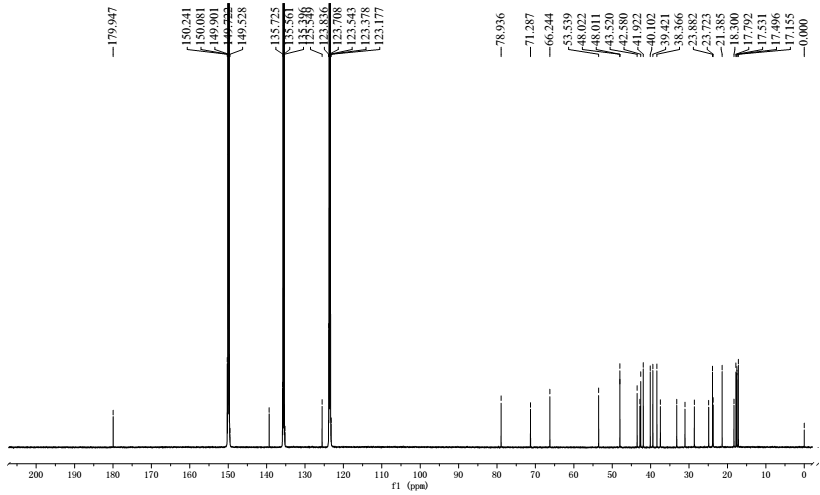


Fig. S11. ^13^C NMR Spectrum of compound **4** (C_5_D_5_N, 150 MHz)


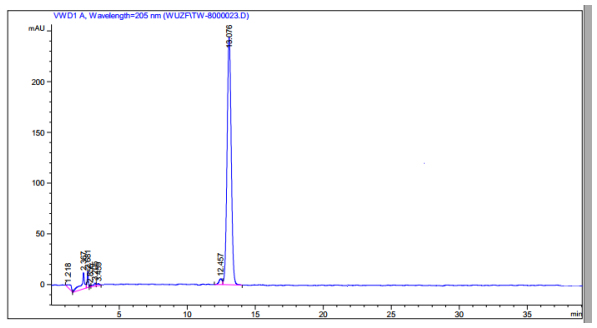


Fig. S12. HPLC profile of compound **4** using UV detection at 205 nm.

**
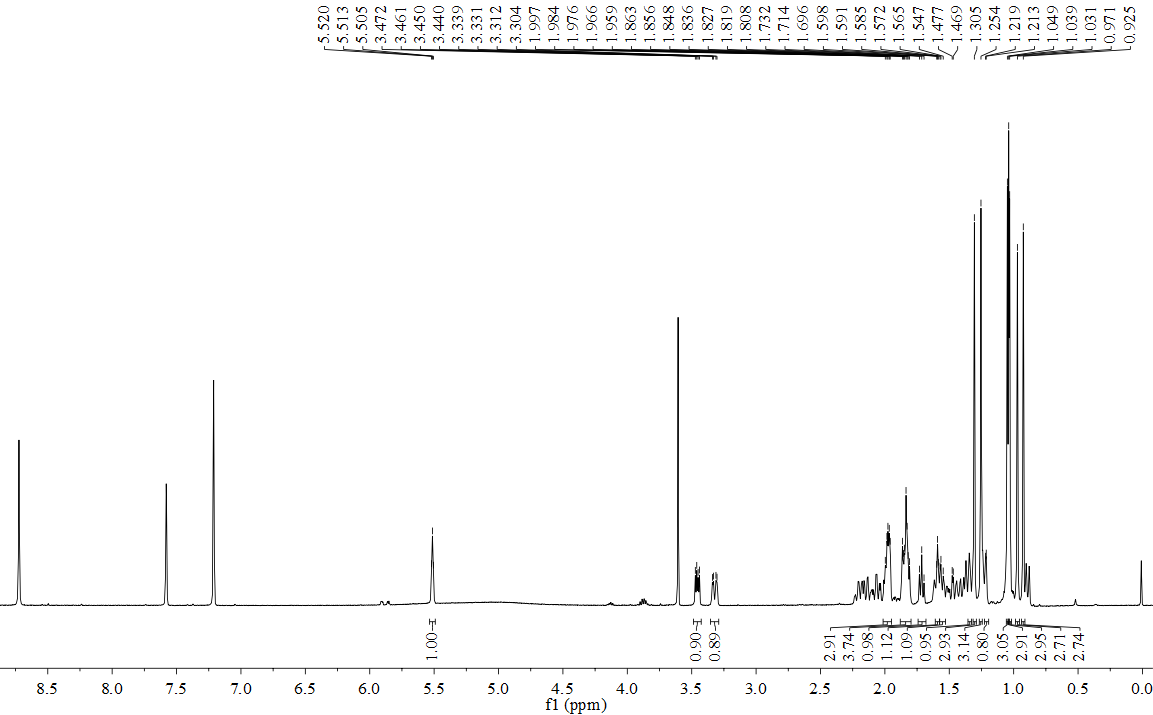
**

Fig. S13. ^1^H NMR Spectrum of compound **5** (C_5_D_5_N, 500 MHz)


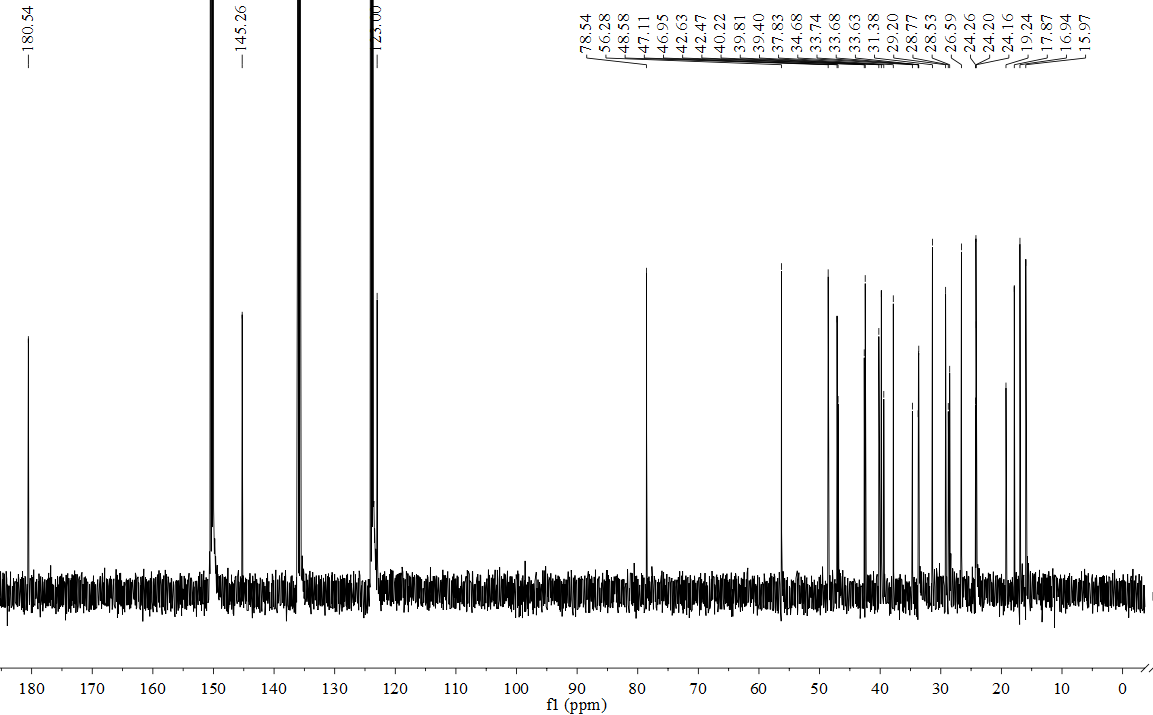


Fig. S14. ^13^C NMR Spectrum of compound **5** (C_5_D_5_N, 125 MHz)

1. ** Laboratory of Translational Medicine, Jiangsu Province Academy of Traditional Chinese Medicine, Nanjing 210028, Jiangsu Province, P.R. China [↑](#footnote-ref-1)
2. * Department of TCMs Pharmaceuticals, School of Traditional Chinese Pharmacy, China Pharmaceutical University, Nanjing 211198, Jiangsu Province, P.R. China

   E-mail addresses: [zjwonderful@hotmail.com](mailto:zjwonderful@hotmail.com) (J. Zhang), [cpu-yzq@cpu.edu.cn](mailto:cpu-yzq@cpu.edu.cn) (Z.Q. Yin) [↑](#footnote-ref-2)
